# High accuracy and learning curve improvement of augmented reality in total knee arthroplasty: A single‐centre study on 157 patients

**DOI:** 10.1002/jeo2.70369

**Published:** 2025-07-13

**Authors:** Giancluca Castellarin, Mattia Sisella, Bernardo Innocenti

**Affiliations:** ^1^ BEAMS Department (Bio Electro and Mechanical Systems), École Polytechnique de Bruxelles Université Libre de Bruxelles Bruxelles Belgium; ^2^ II Unit Orthopaedic Department, Ospedale di Suzzara Mantova Italy

**Keywords:** accuracy, augmented reality, learning curve, total knee arthroplasty

## Abstract

**Purpose:**

Augmented reality (AR) technology is emerging as a viable alternative for improving surgical precision in total knee arthroplasty (TKA). While previous studies have assessed AR accuracy, limited research has explored its learning curve and long‐term performance. This study evaluates the accuracy of an AR‐assisted system in achieving pre‐planned osteotomies and investigates potential improvements over time with increased surgical experience.

**Methods:**

A total of 157 patients underwent TKA using an AR‐based guidance system, with preoperative planning defining varus/valgus and posterior slope angles. The achieved angles were compared to planned values using two error assessment metrics. Additionally, the data from the first 76 procedures were compared to the last 81 to assess a learning curve effect. Surgical time and blood loss were also evaluated.

**Results:**

The AR system maintained high accuracy, with mean deviations of 0.47 ± 0.54° for varus/valgus and 0.76 ± 0.74° for posterior slope. Errors remained below 1° in the majority of cases (98.1% for varus/valgus and 86.0% for posterior slope). Over time, a learning effect was observed, with mean errors decreasing by up to 44.6% for varus/valgus and 8.6% for posterior slope. No significant differences were found in surgery duration or blood loss between the AR and control groups.

**Conclusions:**

This study confirms the reliability of AR‐based guidance in TKA, demonstrating its ability to achieve precise osteotomies with minimal intraoperative variability. Additionally, the results suggest a learning curve effect, with improved accuracy over time. These findings support AR as a promising, cost‐effective alternative to robotic navigation in knee surgery.

**Level of Evidence:**

Level II.

**Abbreviations**
AR: augmented reality
CT: computed tomography
iKA: inverse kinematic alignment
M1: Metric 1
M2: Metric 2
PSI: patient‐specific instrumentation
QR: quick response
TKA: total knee arthroplasty
VR: virtual reality

## INTRODUCTION

Total knee arthroplasty (TKA) is one of the most prevalent procedures in orthopaedic care, with projections indicating an outstanding rise in demand—estimated to grow by 469% in the United States between 2019 and 2060 [23]. Currently, the 10‐year (2012–2023) survivorship rate of about 97% [1] has been reported to be slightly increased compared to previous years. Despite this, the survival rate is not the sole parameter that fully reflects the successful performance of a TKA over its lifespan, as the primary focus should be on the patient rather than the implant itself. The average age for patients undergoing TKA procedures is set at 67.6 ± 9.3 y/o [1]. When coupled with the increasing expectations for a high quality of life, this drives research in the field to develop solutions that prioritize patient needs while ensuring optimal surgical outcomes. Indeed, despite the high survivorship rate, at present, 15%–20% of patients report dissatisfaction following surgery [8, 25]. Key contributing factors may include suboptimal limb alignment, improper implant positioning, soft tissue imbalance or inaccuracies in implant sizing. The adoption of advanced technologies, such as robotic systems and computer‐assisted navigation tools, has become increasingly common to enhance surgical accuracy [13, 21, 22, 31]. In United States, over the past 6 years, the utilization of robotics in primary TKA has increased over sixfold and is now reported in over 15.9% of procedures [1]. However, the adoption of these tools often introduces additional complexities into the surgical workflow, particularly regarding surgeon training and cost‐effectiveness assessments, thereby restricting their utilization to a limited number of surgical centres [7, 11, 20].

A recent introduction to the market is the augmented reality (AR) systems, considered as an alternative approach to tackle the problem described above. Unlike virtual reality (VR), which generates a fictitious world that substitutes the real one [3], AR is capable of augmenting the real world with the superimposition of virtual information [14]. While initially used for spinal surgeries, today, AR systems are widespread in surgical practice, enabling the addition of information by the use of smart glasses worn by the surgeon [12, 14, 24]. These systems are designed to integrate the technical measurement and choices taken during the pre‐planning phase into the field of view, guiding the surgeon during the operation. Along with the utilization of robots and computer‐assisted navigation technologies, the goal of AR systems is to achieve a high level of positioning accuracy during surgery and to diminish errors between planned and performed actions.

Due to its recent introduction in the field of medical applications, the available literature on the use of AR regarding TKA is limited, but constantly growing [17]. This growth highlights the increasing recognition of its importance and effectiveness as a tool. Above all, consistency with pre‐planning targets and operative time are the two parameters mostly investigated [12]. Nonetheless, up to now, studies have focused on a limited number of cases, and no study on the eventual learning effect of the use of AR has been performed.

Therefore, this single‐centre study, which expands the previous published study [6] based on 76 patients, evaluates the effectiveness of an AR‐based system by comparing the discrepancy between the planned preoperative angles, defined by the surgeon, and the achieved cuts. Based on the findings of our previous work, this study was designed to investigate two main hypotheses. First, the aim was to evaluate, on a larger sample size (*N* = 157), whether the AR system provides sufficient accuracy and is suitable for intra‐operative application. Second, it was examined whether the performance of the AR system remains consistent over time, by analyzing surgical data collected over a period of up to 2 years.

## MATERIALS AND METHODS

The protocol selected to conduct this prospective study, was adopted from the previously published one [6], to maintain consistency with previous results. The protocol is presented again in this paper to enhance readability and promote a comprehensive understanding of the procedures. All procedures performed in this study involving human participants were in accordance with the ethical standard of the institutional and/or national research committee and with the 1964 Declaration of Helsinki and its later amendments or comparable ethical standards. All the patients involved in the study were granted their written permission to have their surgical data used for the study.

A total of 161 consecutive patients, from May 2022 to September 2023, were considered as the initial sample. To maintain consistency with the previous study, inclusion criteria were the following: age <85 years and undergoing TKA due to knee osteoarthritis. Exclusion criteria were the following: no neurological or orthopaedic pathologies, no comorbidities or other general issues. Among the patients, 4 were discarded after the software failure emerged during the first calibration of the tool and redirected to a conventional surgical approach, without waiting for the system reinitialization. The final total number of patients included in the sample was 157 (70.4 ± 6.9 y/o; 36.7% males, 63.3% females).

### Surgical technique

The same surgeon (G.C.), in the same hospital centre and adopting the same surgical technique, performed the totality of the surgeries using a medial parapatellar surgical approach in every case, without the use of a tourniquet. To control bleeding, tranexamic acid was administered to each patient, with a dosage of 10 mg per kg of body weight immediately preoperatively and the same dosage 6 h after operation [2].

The selected surgical technique is well established and routinely performed by G.C., besides having already been described in previous publications [4, 5]. All the patients were treated with the same implant design, a Genus MB LS (Adler Ortho). A press‐fit technique was selected for male patients <75 years and female patients <70 years, and in general for all the cases with no specific clinical indication of cement usage. The patella was left untreated and not prosthetized in all patients. The surgical technique used is based on an inverse kinematic alignment (iKA) [27] using conventional instrumentation [16] that utilizes a tibia‐first philosophy trying to maintain as unaltered as possible the patient's joint line [4, 5]. Different from the iKA technique, the femoral cut is executed using the device Extra Medullary Alignment System (Adler Ortho), which includes two distinct and independent spacer paddles aimed at selecting intraoperatively the correct femoral cut inclination, without violating the femoral canal. In line with the current aim of study, the tibial osteotomy was carried out with the assistance of Knee + AR device (Pixee Medical). As reported in the previous study [6], this system requires two quick response (QR) codes optical markers attached to the tibial and femoral bones, tracking the relative positions of the two bodies. Pre‐planned osteotomy angles, both varus/valgus and posterior slope, can be preoperatively recorded in the software based on the X‐rays of the whole leg loaded with partial weight bearing and on the Rosenberg X‐ray projection. After system calibration is performed, the surgeon's view is enhanced by means of real‐time information provided via smart glasses connected to the AR system. For this study, a personalized cutting block, specifically designed for the purpose of the implant manufacturer, is employed and can be moved until it matches the planned osteotomy parameters. After cut executions, the resulting angles (both varus/valgus and posterior slope) were measured using a dedicated goniometer (accuracy of 0.5°) and compared postoperatively with the planned ones. A control group (*N* = 100), previously treated with the same protocol except for the use of the AR system, was considered as reference for the bleeding and surgery time comparison. Moreover, average bleeding was measured as volume of blood present in the drainage bag after its removal, whereas mean AR system usage time was calculated as the difference between the total surgical time of the two groups of patients. To assess the potential learning curve effect, the error in varus/valgus and posterior slope was compared between two nearly homogeneous groups: the first 76 patients and the last 81 patients.

### Data analyzes

A power analysis was conducted using GPower 3.1.9.7 [9] to estimate the achieved power. The analysis was based on an *α* level of 0.05 (commonly accepted in the literature), an effect size calculated from the mean and standard deviation values reported in Table 1 for slope and varus angles, and a total sample size of 157. For both slope and varus, the statistical power of the study exceeded 0.95.

**Table 1:** Mean, SD and ranges of the differences between the angles AR achieved and the planned ones.

Angles	Mean	SD	Range
Varus/valgus	0.47°	0.54°	From 0° to 2°
Posterior slope	0.76°	0.74°	From 0° to 3°

Abbreviations: AR, augmented reality; SD, standard deviation.

Pre‐surgery (planned) and post‐surgery (achieved) varus/valgus and posterior slope angles were recorded for each patient and compared to compute the final difference for each case. To avoid any potential compensation effect, such variation was expressed as absolute values, and a Pearson test was performed to assess the normal distribution of the values. Two different metrics were adopted to evaluate the performance and tendency to error of the AR system. For the first metric (M1), deviations were stratified according to the magnitude of the obtained error, separately for varus/valgus and posterior slope. This led to 3 categories of errors (0°, 1° and 2°) for the varus/valgus cut and four categories (0°, 1°, 2° and ≥2°) for the posterior slope cut. This stratification was employed to identify the magnitude of the most frequent error. On the other hand, the second metric (M2) adopted a categorization based on pre‐planned angles, to detect eventual target angles more prone to error and therefore analyze the accuracy of the AR system in function of the magnitude of the cut angle to perform. M2 led to three categories (0°, 1° and ≥2°) for the varus/valgus and four categories (≤2°, 3°, 4° and ≥5°) for the posterior slope. Finally, by dividing the sample into two groups—one linked to the previously mentioned study and the other to the current extended study with data from new patients—a potential error reduction between the first 76 patients and the last 81 was analyzed, to highlight the presence of learning curve for the AR system.

## RESULTS

After performing the Pearson normality test, it has been proved that the collected data followed a normal distribution (*p* > 0.05), and the sample size of 157 patients aligned with the normal curve.

The AR device's total usage time was 5 ± 1 min for all the patients, with no difference between the first and second groups. The blood loss volume was registered similarly to the previous study, and no statistical differences can be highlighted between the values for the AR group and those of the control group.

Considering all the AR‐treated patients, it emerges that the varus/valgus angle diverged from the pre‐planned angle by 0.47 ± 0.54°, while the posterior slope angle diverged by 0.76 ± 0.74°. The value range of the deviations was 0–2° and 0–3°, respectively, for varus/valgus and posterior slope angle. These results are reported in Table 1.

Focusing on the metric M1, almost the totality of the performed varus/valgus cuts (98.1%) were associated with no error (0°) or an error equal to or below 1°. In particular, 54.8% (86 out of 157) of the patients received a correct cut (0°) and 43.3% (68 out of 157) a cut affected by a 1° error. The remaining cuts were performed with a difference of 2°, and covered only 1.9% (3 out of 157) of the total.

For the posterior slope angle cuts, the overall trend is similar, with 40.1% (63 out of 157) of them non‐affected by errors, 45.9% (72 out of 157) with 1° of error, 12.1% (19 out of 157) with 2° of deviation and 1.9% (3 out of 157) affected by errors of ≥2°. These results are also reported in Table 2.

**Table 2:** Categorization with metric M1 for varus/valgus and posterior slope cuts.

	Varus/valgus			Posterior slope			
Error	0°	1°	2°	0°	1°	2°	≥2°
N	86	68	3	63	72	19	3
%	54.8	43.3	1.9	40.1	45.9	12.1	1.9

**Figure 1:**
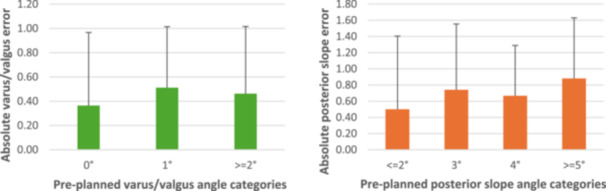
Mean absolute varus/valgus (left) and posterior slope (right) errors (°) for different varus groups.

**Figure 2:**
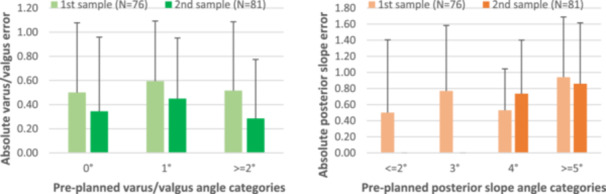
Comparison between the first (*N* = 76) and second (*N* = 81) samples in terms of mean absolute varus/valgus (left) and posterior slope (right) errors (°) for different varus groups.

Considering the metric M2, for the varus/valgus cut (Figure 1a), the higher number of pre‐planned angles belongs to the 1° category (54.8%, 86 out of 157), while 0° and ≥2° almost equally share the repartition of the remaining cuts (respectively 21.0%, 33 of 157 and 24.2, 38 of 157). For these three categories (0°, 1 and ≥2°), the associated error is equal to 0.36 ± 0.60°, 0.51 ± 0.50° and 0.46 ± 0.55°, respectively. Regarding posterior slope angles (Figure 2b), as mentioned before the four categories are ≤2°, 3°, 4° and ≥5°, and the most populated group is the last one: 7.6% (12 out of 157), 17.2% (27 out of 157), 32.5% (51 out of 157) and 42.7% (67 out of 157), respectively. The calculated deviations are the following: 0.50 ± 0.90°, 0.74 ± 0.81°, 0.67 ± 0.62° and 0.88 ± 0.75°, respectively. Therefore, regardless of the considered category, both varus/valgus and posterior slope cuts, the error between the pre‐planned and the executed angle is always lower than 1° on average. This is also indirectly reflected in the results calculated with M1.

Finally, looking at the comparison between the data set of the previous study and the sample made by the newly included patients, a decrease in errors can be observed through M2, mostly in varus/valgus (Figure 2a) and slightly in posterior slope angles (Figure 2b). Specifically, better performance results from lowered mean errors of 31.0%, 24.5% and 44.6%, respectively, for the three categories of varus/valgus cuts. As for the posterior slope, the stratifications of the first and the second samples did not return the same categories. Nonetheless, for the ≥5° group, the mean error decreased by 8.6%, whereas only for the 4° group an increase occurred, equal to 38.9%.

## DISCUSSION

The findings of this study provide further insights into the accuracy, effectiveness and learning curve of an AR‐based system for assisting TKA. The deviation between pre‐planned and performed angles remained consistently low for both varus/valgus and posterior slope directions, with average discrepancies of 0.47 ± 0.54° and 0.76 ± 0.74°, respectively. These values indicate that the AR system can support surgeons in achieving their targeted cuts with a level of accuracy that minimizes potential biomechanical alterations postoperatively, maintaining an overall error below 1°.

Another important result is related to the repeatability of the system, as the data analyzed through M1 demonstrated that the vast majority of varus/valgus cuts were either perfectly executed or deviated by only 1°, with only a minor proportion (1.9%) exceeding this threshold. Similarly, posterior slope cuts followed a comparable pattern, with 40.1% executed with no error, 45.9% with a 1° deviation, and only 1.9% exceeding 2° of error. This consistency in results suggests again that the AR system offers a reliable and repeatable intraoperative assistance tool capable of maintaining high levels of accuracy across different surgical cases.

Considering the initial preplanned values, indicative of a patient deformity, the analysis through metric M2 revealed that deviations remained below 1° across all categories, irrespective of the initial pre‐planned target angles. These findings confirm the AR system's robustness in maintaining precision independently of the patient's anatomy and the planned osteotomy inclination, which is a crucial factor for ensuring reproducible surgical cuts.

Therefore, the system's ability to provide real‐time feedback through smart glasses, coupled with the use of QR markers for bone tracking, appears to contribute significantly to maintaining alignment and reducing intraoperative variability.

Another important aspect examined in this study was related to the learning curve of the use of such tool, evaluated by comparing the first 76 patients, obtained from the original data set from the previous research [6] and the last 81 patients that compose the overall data set presented here. The decrease in errors observed in the later treated patients suggests an existing potential learning curve associated with the adoption of the AR system. Specifically, reductions in mean errors of up to 44.6% for varus/valgus and 8.6% for posterior slope cuts indicate that increased experience with the system may lead to further refinement in surgical precision. Despite an increase in error in one group and the non‐statistical difference in the compared values (varus/valgus: 0° *p* = 0.318; 1° *p* = 0.093; 2° *p* = 0.165; posterior slope: 4° *p* = 0.135; 5° *p* = 0.351), these results highlight the potential for enhanced outcomes as familiarity with this technology increases. Furthermore, examining this aspect also highlights the robustness of the results, which remain consistent with the previous study, thereby suggesting the overall reliability of the system.

Therefore, the results of the present study confirmed the first hypothesis, supporting the potential clinical relevance of the system. In contrast, the second hypothesis was not confirmed, as the data revealed a trend consistent with a learning curve. Although not statistically significant, this trend suggests that surgical performance improves with continued use of the system.

AR systems offer several advantages over both general robotic systems and patient‐specific instrumentation (PSI) in orthopaedic surgery. Unlike robotic‐assisted procedures, AR does not require preoperative computed tomography (CT) scans, which represent a significant cost burden, whether covered by the patient or the healthcare provider. Furthermore, AR systems typically do not increase surgical time and avoid the need for complex preoperative planning. Instead, they allow for a planning process that closely mirrors standard surgical techniques, contributing to a smoother integration into existing clinical workflows. Another important benefit is the reduced cost, as AR does not require the dedicated hardware infrastructure associated with robotic systems. Compared to PSI, AR also presents notable benefits. As previously mentioned, the elimination of preoperative CT imaging simplifies the preparatory phase and reduces associated costs. Additionally, AR systems do not require the 2‐ to 3‐week lead time typically needed for the design, production and delivery of custom‐made cutting blocks, thus alleviating logistical challenges and minimizing the risk of administrative errors. This is particularly relevant in high‐volume surgical centres performing multiple procedures daily, where the coordination and management of PSI can be burdensome. Although surgical times with AR are comparable to those with PSI, the overall cost is lower. Most AR components are reusable, with the exception of the sensors, which are considerably less expensive than the single‐use custom blocks required for PSI.

When comparing the results of the present study to those found in the existing literature, it is clear that the AR system provides a level of accuracy that is consistent with, if not superior to, other technologies currently available for TKA. For instance, studies examining the use of robotic‐assisted surgery in TKA demonstrate a clear improvement in the results in comparison with standard techniques, with deviations between 1.5° and 2° in osteotomy alignment [21, 22], while the current study demonstrates a lower mean deviation of 0.47° for varus/valgus and 0.76° for posterior slope angles, similarly to other studies [26, 29]. Yi et al., using robotic surgery on eight cadaveric knees, have identified an average error of 0.88 for varus/valgus, which is similar to the results of the current paper, even if considering the surgery on a real patient. Moreover, the proposed results are promising if compared with the study of Yee et al. [28], which by comparing the accuracy of image‐free vs image‐based robotic systems, identifies an error for varus/valgus cut of 1.16° and 1.35° and 1.31° and 2.66° for posterior slope cut, respectively. This suggests that AR systems may offer a possible more accessible, cost‐effective alternative to robotic systems, with comparable or even not‐inferior accuracy in guiding surgical cuts. Furthermore, the results align with findings from studies utilizing computer‐assisted navigation systems. For example, a study by Zhang et al. [31] reported that computer‐assisted navigation reduced alignment errors to about 1° on average. Zorman et al. [32] identified on 72 patients an accuracy of 0.4° in the varus/valgus absolute error with the use of navigation compared to 1.85° without, even if unfortunately no data on the variability of the tibial slope is quantitatively reported. Graydon et al. [10] measured, on an artificial leg phantom, a range of error of 0.8° and 1.5° for varus/valgus and posterior slope cut, respectively. Similarly, Pitto et al. [19] identified similar values with a varus/valgus and posterior slope variability equal to or lower than 1° using an artificial phantom leg. However, the AR system in the present study consistently achieved similar or not‐inferior outcomes, analyzing the real clinical use in patients, without introducing the additional complexity and cost associated with robotics or other high‐tech systems. The ease of use, with the real‐time integration of pre‐planned angles into the surgeon's field of view through smart glasses, appears to provide a practical advantage over other methods that require more bulky equipment or extensive training.

A comparison with previous studies that employed AR technology in TKA also shows that the tool's performance continues to improve with increased experience. For instance, the results from this extended data set suggest a potential learning curve, as indicated by the maximal reductions of 44.6% in errors for varus/valgus cuts and an 8.6% for posterior slope cuts over the course of the study. This pattern is similar to that observed in earlier research involving AR in TKA, where familiarity with the system contributed to improved accuracy and efficiency [12]. The slight improvement in performance as surgeons gain experience with the technology further emphasizes the system's potential for refinement and optimization in clinical practice. Looking at surgical active robot, Mahure et al. [18] demonstrated a short learning curve on 10–20 cases, mainly associated with the surgical time dedicated to the robotic‐specific portion of the case; no learning curve‐associated device‐related complications, three‐dimensional component position, or patient‐reported outcome scores were reported. The current study, considering the first 76 cases and the latter 81, shows for AR a different trend, with a learning curve more focused on the reduction of variability but not on time. This last conclusion could be justified as our study's numerosity for the first study is based on 76 patients, while the study of Mahure et al. analyses the first 10–12 patients in comparison with the remaining 100. The results of Mahure et al. are also similar to those of Kayani et al. [15], stating the robotic‐arm assisted TKA has a learning curve of seven cases for integration into the surgical workflow, but no learning curve effect for accuracy of implant positioning. Moreover, Zhang et al. [30] determined that the learning curve for robotic‐assisted TKA ranged from 8 to 20 cases, with no observable learning curve effect detected for bone‐cutting accuracy or limb alignment.

Despite these promising results, certain limitations must be acknowledged. First, the study was conducted within a single centre and involved a single experienced surgeon performing all procedures. This standardization ensured consistency in technique but may limit the generalizability of findings to broader surgical practices with varying levels of expertise. Additionally, the use of a single prosthetic model and a fixed surgical approach means that further investigations are required to evaluate the AR system's adaptability to different implant designs and surgical techniques. Furthermore, this study considered only two parameters, excluding tibial cut thickness, an essential factor that plays a crucial role in guiding the TKA procedure.

Finally, the economic feasibility of implementing AR‐based guidance systems in routine TKA procedures remains an area of debate. While these systems offer tangible benefits in terms of surgical accuracy and cost, a precise cost analysis would be necessary to definitively establish the actual savings.

## CONCLUSION

In summary, this study confirms the effectiveness of AR‐assisted TKA in achieving highly accurate osteotomies, with minimal deviation from pre‐planned surgical targets. The system demonstrated consistent precision across different planned angles and showed potential for improvement with increased user experience. Moreover, the study highlights that the varus and slope cut angles obtained with the AR‐based system closely matched the preplanned configuration, with errors consistently below 1° regardless of the target value.

Additionally, the minimal impact of using this system on overall surgery duration and relative patient bleeding suggests that AR‐based assistance does not introduce significant intraoperative inefficiencies. These findings indicate that AR‐assisted surgery represents a promising tool that surgeons should consider when selecting navigation technologies for TKA. Finally, the analysis of the learning curve suggests that increased experience with the AR system leads to a reduction in surgical errors, particularly in varus/valgus cuts, where a maximal decrease of 44.6% in mean errors was observed, and to a lesser extent in posterior slope cuts, with an 8.6% reduction. These findings indicate that while the system ensures high accuracy, continued use may further refine surgical precision, reinforcing its potential for clinical optimization.

While the results are promising, further multi‐centre studies and long‐term follow‐ups are necessary to fully assess the clinical benefits and economic viability of AR technology in orthopaedic surgery. Future research should also explore the integration of AR systems into diverse surgical workflows to optimize their applicability and accessibility across different healthcare settings.

## AUTHOR CONTRIBUTIONS

*Conceptualization*: Gianluca Castellarin and Bernardo Innocenti. *Methodology*: Gianluca Castellarin, Mattia Sisella and Bernardo Innocenti. *Formal analysis and investigation*: Gianluca Castellarin, Mattia Sisella and Bernardo Innocenti. *Writing—original draft preparation*: Mattia Sisella. *Writing—review and editing*: Mattia Sisella and Bernardo Innocenti. *Funding acquisition*: N/A. *Resources*: Gianluca Castellarin and Bernardo Innocenti. *Supervision*: Gianluca Castellarin and Bernardo Innocenti.

## CONFLICT OF INTEREST STATEMENT

The authors declare no conflicts of interest.

## ETHICS STATEMENT

All the patients involved in the study granted their written permission to have their surgical data used for the study.

## Data Availability

Additional data can be provided upon request.
